# High perceived energy: exploring distinct patterns of energetic and cognitive functioning in older adults

**DOI:** 10.3389/fpsyt.2026.1872379

**Published:** 2026-07-15

**Authors:** Orsola Marra, Diego Primavera, Giulia Cossu, Alessandra Perra, Michela Atzeni, Elisa Pintus, Massimo Tusconi, Gianluca Castelnuovo, Maria Veronica Brasesco, Gustavo Mausel, Mauro Giovanni Carta

**Affiliations:** 1 Department of Medical Sciences and Public Health, University of Cagliari, Cagliari, Italy; 2 PhD Program in Capacities Building for Global Health, University of Cagliari, Cagliari, Italy; 3 Department of Medicine and Surgery, University “Kore” of Enna, Enna, Italy; 4 Association of the European University of the Mediterranean, Cagliari, Italy; 5 Center for Liaison Psychiatry and Psychosomatics, University Hospital of Cagliari, Cagliari, Italy; 6 Department of Psychology, Catholic University of Milan, Milan, Italy; 7 IRCCS Istituto Auxologico Italiano, Clinical Psychology Research Laboratory, Milan, Italy; 8 Facultad de Humanidades, Universidad del Museo Social Argentino, Buenos Aires, Argentina

**Keywords:** cognitive performance, depressive symptoms, hyperenergy, quality of life, social and behavioral rhythm regulation

## Abstract

**Background:**

A hyperthymic temperament and high perceived energy are often framed as positive dispositional traits, particularly in later life. Indeed, these characteristics are generally associated with a greater tendency to preserve autonomy and with more favorable outcomes in terms of health, engagement, and participation in community life-key dimensions of successful aging. However, the relationship between elevated perceived energy and cognitive performance in older adults remains insufficiently explored. Therefore, this exploratory study aimed to compare cognitive performance and well-being in older adults with different self-reported levels of subjective energy, and to explore potential phenotypic heterogeneity within this population.

**Methods:**

We conducted a cross-sectional study on older adults from the general population. Participants were classified as having higher versus lower levels of perceived energy based on item 10 of the Short Form Health Survey (SF-12) questionnaire. Cognitive performance was assessed using the Addenbrooke’s Cognitive Examination-Revised (ACE-R). Depressive symptoms were evaluated with the Patient Health Questionnaire-9 (PHQ-9), biological and behavioral rhythm regulation with the Biological Rhythms Interview of Assessment in Neuropsychiatry (BRIAN), and quality of life with the SF-12 were also evaluated to characterize overall well-being.

**Results:**

Statistically significant difference emerged in global cognitive performance between individuals with higher versus lower perceived energy (ES ACE-R p=0.012). Although subscale scores were within the normative range in both groups, individuals with higher perceived energy consistently showed significantly lower scores in language (p = 0.011) and attention/orientation (p = 0.049) compared to the lower perceived energy group. Interestingly, the higher perceived energy group also reported fewer depressive symptoms (p = 0.013), better social and behavioral rhythm regulation (p = 0.005), and higher quality of life (p < 0.001).

**Discussion/Conclusions:**

Hyperenergy in older adults with preserved well-being is not associated with global cognitive impairment, but rather with subtle domain-specific performance variations, suggesting a heterogeneous underlying profile. These findings support a high-energy profile that does not appear to be associated with functional impairment, but is instead characterized by preserved or enhanced functioning and overall well-being, despite selective areas of lower performance. Longitudinal studies are needed to clarify its clinical and cognitive implications.

## Introduction

Cognitive deficits are often considered a core feature of bipolar disorder, affecting multiple domains such as attention, memory, and executive functioning (1–3). These impairments are observed not only during acute mood episodes (mania or depression), but often persist during euthymic (remitted) phases, suggesting they are partly trait-like rather than purely state-dependent (4, 5). The most consistently reported deficits involve verbal memory, working memory, attention, and executive functions (6). Importantly, these impairments are associated with poorer functional outcomes, including occupational and social difficulties (7). Although some patients recover cognitively after acute episodes, a substantial proportion show persistent deficits even in remission, and patients themselves may not accurately perceive objective impairments (8). It is certain, however, that not all patients with bipolar disorder present cognitive deficits (1). In people with bipolar disorder in remission, three distinct groups can be distinguished for cognitive functioning: with intact performance, with selective cognitive impairment, and with global cognitive impairment (8–10). It has been observed that the differences remain unchanged over a long period of observation (11). It should be noted that in the high-performance group, cognitive functioning remained intact; this could therefore define a specific group (11).

Recent literature highlights that non-pathological, subthreshold forms of hyperactivity exist along a continuum from normal variability to clinical disorders. Hyperthymic temperament, characterized by stable high energy, reduced need for sleep, and goal-directed activity, often represents a non-pathological, adaptive phenotype associated with resilience and reduced clinical severity (12). In contrast, when hyperactivity co-occurs with circadian dysregulation and depressive symptoms, it may reflect a subthreshold condition akin to DYMERS (13).

In the present study, high perceived energy is conceptualized as a dimensional construct reflecting individual differences in subjective energy levels and vitality. This perspective aligns with the use of item 10 of the SF-12 (14, 15) in previous population-based studies (16), where it has been employed as a brief indicator of perceived energy rather than as a measure of psychopathological activation or hyperactivity. Such energy-related indicators may be associated with broader dimensions of affective and behavioral regulation and therefore may be useful for exploring variability in non-clinical populations (16).

Although elevated energy levels have historically been discussed within broader psychopathological frameworks, including hyperthymic temperament and bipolar-spectrum models (17, 18) the present study does not assess these constructs directly. They are mentioned only as part of the conceptual background supporting the investigation of energy-related dimensions across a continuum extending from normal variation to clinical conditions.

However, very recently, an adaptive increase in energy and activation has been described, observed particularly in gifted individuals facing a challenging and stimulating task. The importance of self-regulation has been highlighted in relation to both performance success and recovery processes (19), particularly in the context of sport and athletic performance. In other words, it refers to a highly developed capacity to manage and modulate energy, with alternating phases of activation and recovery that are particularly functional for achieving performance goals (20).

This ability can be improved with training, but “baseline” characteristics are important. Indeed, previous studies have identified groups of older adults characterized by elevated levels of activity, novelty seeking, and perceived energy who also showed a higher frequency of genetic variants previously associated with bipolar disorder (21–23). This raises the interesting question of whether, despite this genetic predisposition, these individuals might maintain normal cognitive functioning, potentially through compensatory strategies (24–27). More broadly, dimensional models of mood and energy regulation suggest that variations in activation and energy expenditure may be relevant across a continuum extending from normal individual differences to clinically significant conditions (28–30). Within this framework, exploring the relationship between perceived energy and cognitive functioning in older adults may provide useful insights into the heterogeneity of energy-related phenotypes and their potential implications for cognitive aging.

Against this background, it is of interest to explore whether older adults reporting higher levels of subjective energy differ from those reporting lower levels of energy in terms of cognitive functioning and well-being. Although elevated subjective energy may be associated with favorable indicators such as resilience, engagement, and preserved functioning, its relationship with cognitive performance remains poorly understood.

The present exploratory study therefore aimed to compare cognitive performance, depressive symptoms, social and behavioral rhythm regulation, and quality of life between older adults reporting higher versus lower levels of perceived energy. Given the exploratory nature of the study and the limited operationalization of the construct, all interpretations should be considered hypothesis-generating rather than confirmatory.

## Methods

### Sample and design

The study sample consisted of community-dwelling adults aged 65 years and older, with no gender restrictions, recruited from a population-based cohort. Based on research instruments, the study was divided into participants with higher versus lower levels of perceived energy (independent variable). Cognitive performance (dependent variable) was assessed in the two samples. The two samples were also compared with respect to depressive symptom frequency, social e behavioral rhythm regulation, and quality of life.

### Study tools

To distinguish the level of hyperenergy in the sample, item 10 of the SF-12 questionnaire was used. This item captures the frequency with which individuals perceive themselves as feeling full of energy in recent weeks and was used as a proxy indicator of energy-related activation. Notably, it has been previously used as a validated brief screening indicator in large cohorts for identifying individuals with elevated levels of energy and behavioral activation (16, 31). Although a single-item measure cannot fully represent the complexity of this construct, it may offer a practical means of capturing relevant variability. For example, the previously observed association (16) between SF-12 item 10 and Mood Disorder Questionnaire (MDQ) positivity suggests a partial convergence with domains of activation, energy, and activity; however, it does not adequately capture the psychopathological hyperactivity or bipolar-spectrum activation for which the MDQ was originally developed. Based on the responses, the sample was divided individuals with higher levels of perceived energy (answers 6 to 10 to item 10 of the SF-12) and participants with lower levels of perceived energy (answers 1 to 4 to item 10 of the SF-12). Participants who selected the intermediate response option (score = 5) were excluded to reduce classification ambiguity and to increase the contrast between groups. This approach was adopted to minimize potential overlap between adjacent levels of perceived energy and to improve the interpretability of group comparisons within an exploratory design.

1. The frequency of depressive symptoms in the two samples was assessed using the Patient Health Questionnaire-9 (PHQ-9) (32, 33) in the Italian version already validated (34). This is a self-assessment scale widely used as a screener to identify depressive episodes and to measure symptom frequency. A cut-off of 8/9 identifies people with a high probability of having a depressive episode of at least moderate level (33).
2. Social and behavioral rhythms in the samples were measured using the Italian version of the Biological Rhythms Interview of Assessment in Neuropsychiatry (BRIAN) scale (35). The 18 items assessed sleep (specifically difficulty falling asleep, problems waking up, being satisfied with sleep time), social activities (problems completing usual activities), social rhythm (problems interacting with people and/or excessive use of TV or internet), problems respecting eating habits (meal times and quantity of food), and whether the dominant rhythm is nocturnal or diurnal. All items were evaluated using a 4-point Likert scale ranging from 1 = not at all to 4 = always. The observation time of the instrument refers to the 14 days preceding the evaluation. High scores indicate dysfunctional rhythms.
3. The SF-12 (14, 15), adopted in the Italian version, previously validated (14, 36), was used to measure Health Related Quality of Life in the two samples. SF-12 is a self-report questionnaire that assesses two distinct dimensions (physical and mental health). The score ranges from 12 to 47; higher scores indicate a higher level of Health Related.
4. Cognitive functions were assessed using the Addenbrooke’s Cognitive Examination (ACE-R), a version evaluated in Italian (37). This is a brief global cognitive screening instrument (approximately 15–20 minutes in people without cognitive impairment). The ACE-R assesses five cognitive areas: attention/orientation, memory, verbal fluency, language, and visuospatial skills. The maximum score is 100; a score of 66 or less indicates mild cognitive impairment. While it covers multiple cognitive domains, it does not provide a detailed domain-specific neuropsychological evaluation comparable to a comprehensive battery. Accordingly, in the present study, the ACE-R was used for screening purposes rather than for an in-depth assessment of specific cognitive domains.

### Statistical analysis

Nominal data were analyzed using the chi-square test, with Yates’ correction when required (e.g., comparisons of frequency by sex across the two study samples). The numerical data were analyzed using one-way analysis of Variance with Bonferroni correction. The statistical analyses were conducted using Stata software, version 17.0 (38).

### Ethics

The study was approved by the Independent Ethical Committee of the “Azienda Ospedaliero Universitaria di Cagliari” (University Hospital of Cagliari), on September 30, 2020, under protocol NP/2022/2893 and its subsequent amendments. All study protocols were conducted in accordance with the Declaration of Helsinki (39). All participants signed an informed consent.

## Results

**Table 1** presents the characteristics of the two samples, comparing sex, age, education level, well-being, self-regulation, and depressive symptoms by sex. The two samples compared are perfectly balanced by age and sex; the sample of older adults with high perceived energy (Item 10 SF-12 = 6) has a slightly higher frequency of graduates (36.4% vs. 25.8%), but the difference is not statistically significant. On the other hand, the high perceived energy group appears to have fewer depressive symptoms (PHQ score 2.27 ± 2.52 vs. 5.37 ± 3.84; p=0.013), a more regular rhythm (Brian score 28.00 ± 4.15 vs. 33.91 ± 6.46; p=0.005), and a better perception of quality of life (SF-12 score 40.36 ± 3.31 vs. 32.01 ± 4.83).

**Table 1:** Characteristics of the two samples compared by sex, age, education levels, quality of life, social and behavioral and depressive symptoms.

Item	Sex (female) N (%)	Age mean ± SD	Graduated or more N (%)	PHQ-9 mean ± SD	BRIAN mean ± SD	SF-12 mean ± SD total score
High perceived energy (Item 10 SF-12 = 6)	7 (63.6%)	74.18±3.48	4 (36.4%)	2.27±2.52	28.00±4.15	40.36±3.31
Low perceived energy (Item 10 SF-12=1- 4)	36 (62.1%)	75.17±4.74	15 (25.8%)	5.37±3.84	33.91±6.46	32.01±4.83
Comparison between sample shown by Chi-Square (χ^2^) and Fisher’s F tests	χ^2^ 1df (Yates corrected) =0.001 p=0.999	F (1,67 df) =0.433 p=0.513	χ^2^ 1df (Yates corrected) =0.120 p=0.729	F (1,67 df) =6.586 p=0.013	F (1,67 df) =8.422 p=0.005	F (1,67 df) =30.010 p<0.001

Data are presented as frequencies (percentages, %) for categorical variables and as means ± standard deviations (SD) for continuous variables. BRIAN, Biological Rhythms Interview of Assessment in Neuropsychiatry; *F(df)​*, Fisher’s F-ratio (with specific degrees of freedom in subscripts); PHQ-9, Patient Health Questionnaire-9; SF-12, 12-item Short Form Survey; *χ^2^(df)*, Chi-Square test with Yates’ continuity correction (with specific degrees of freedom in subscripts).

**Table 2** shows the cognitive performance in the two samples; the sample older adults with high perceived energy (Item 10 SF-12 = 6) showed a lower “standardized” total ACE score than the control sample, but without reaching a difference of statistical significance (83.97 ± 10.79 vs 86.81 ± 6.25; p=0.192). However, slightly lower scores with statistically significant differences emerged for the high-energy subgroup in specific areas, namely: Attention and Orientation (16.90±2.23 vs 17.67±0.87; p=0.049); in the language subscale (23.27 ± 4.61 vs 25.03 ± 1.08; p=0.011); and considering the score in four classes of functionality (2.81 ± 1.26 vs 3.58 ± 0.83; p=0.012). The score on the Memory scale was lower in the hyperenergy group, with a difference that bordered on statistical significance (20.27±6.08 vs 22.63±3.76; p=0.091). In all seven assessments, the hyperenergy sample showed lower scores, although on the Memory, Verbal Fluency, and Visual-spatial skills scales, the difference did not reach statistical significance.

**Table 2:** Cognitive performance in the two samples.

Item	High perceived energy (n=11) (Item 10 SF-12 = 6)	Low perceived energy item 10 SF-12 =1-4) (N = 58)	Statistics ANOVA 1-way (1, 67 df)
SC ACE-R (total)	83.97±10.79	86.81±6.25	F=1.727; p=0.192
Attention and Orientation	16.90±2.23	17.67±0.87	F=3.965; p=0.049
Memory	20.27±6.08	22.63±3.76	F=2.935; p=0.091
Verbal fluency	9.27±3.24	10.34±2.46	F=1.576; p=0.214
Language	23.27±4.61	25.03±1.08	F=6.878; p=0.011
Visual-spatial skills	13.90±1.83	14.34±1.66	F=0.629; p=0.430
ES	2.81±1.26	3.58±0.83	F= 6.661; p=0.012

Data are presented as means ± standard deviations (SD). ACE-R, Addenbrooke’s Cognitive Examination-Revised; ES, Equivalent Score; F (df1, df2), Fisher’s *F*-statistic from One-Way Analysis of Variance (ANOVA), where *df1* and *df2* represent the respective degrees of freedom; P, indicating statistical significance; SC, Standardized/Corrected Score.

## Discussion

The present findings provide a nuanced perspective on the relationship between hyperenergy and cognitive performance in older adults. Consistent with the initial hypothesis aimed at examining potential differences in cognitive performance, the results of the present study indicate that individuals with high perceived energy exhibited lower cognitive performance compared to those with lower activity levels. However, it is important to note that the high perceived energy group remained fully within normative ranges, with no evidence of global cognitive impairment. Moreover, this group demonstrated preserved and in some domains enhanced quality of life, more effective regulation of social and behavioral rhythms, and fewer depressive symptoms.

However, a consistent pattern emerged across the results. Individuals with high perceived energy showed systematically lower scores in all cognitive domains, with statistically significant differences in specific areas, particularly language and attention/orientation, as well as in functional classification. This profile points to selective inefficiencies rather than a generalized deficit (40).

Given the exploratory nature of this study one preliminary interpretation concerns differences in cognitive processing style. Individuals with high perceived energy may preferentially rely on rapid, global, and “synthetic” processing strategies, favoring speed, integration, and goal-directed behavior over detailed and sustained analytical processing. While this style may be advantageous in many real-life contexts, over time it could lead to a relative under-engagement of attention-demanding and analytically oriented functions, particularly those involving sustained attention, orientation, and fine-grained language processing. This interpretation is consistent with previous findings linking affective temperaments to specific cognitive profiles (41). In line with Fuzzy Trace Theory (42), these individuals may rely more on gist processing than verbatim processing, achieving adequate overall performance but slightly lower scores in tasks that require sustained attention or precise verbal processing. This highlights potential differences in cognitive style rather than absolute cognitive capacity (43, 44).

Furthermore, neuropsychological tests capture performance outcomes but do not reflect the underlying strategies used; therefore, the observed lower cognitive performance in certain domains may plausibily reflect different cognitive approaches rather than a reduction in cognitive capacity (45).

A second key element is the descriptive trend toward greater score dispersion observed in the high energy group, reflected in the larger standard deviations across cognitive measures. While this descriptive pattern cannot be formally interpreted as statistical variance heterogeneity due to our sample constraints, it tentatively raises the question of whether high perceived energy constitutes a homogeneous condition or rather a heterogeneous construct encompassing distinct underlying profiles (46).

Within this framework, the substantial variability observed within the high-energy group raises the possibility that elevated perceived energy may not represent a homogeneous condition. Rather, it may reflect the coexistence of different underlying profiles, ranging from potentially advantageous forms of high activation to more vulnerable patterns of functioning. Importantly, the present study did not directly assess DYMERS or related psychopathological constructs. Therefore, any reference to DYMERS-like profiles should be considered speculative and hypothesis-generating. Recent contributions have suggested that energy regulation and dysregulation may be conceptualized along a continuum extending from non-clinical variability to clinically relevant conditions (13, 47, 48), but further studies using dedicated assessments are required to explore this possibility.

The descriptive variability observed in cognitive performance may indicate that individuals with similar levels of perceived energy do not necessarily share the same cognitive profile. Although the present sample is too small to formally identify distinct subgroups, the observed dispersion raises the possibility that different cognitive trajectories or patterns of functioning may coexist within high-energy populations. In this sense, perceived energy may act as a marker of heterogeneity rather than as a uniform characteristic, encompassing both potentially favorable and more vulnerable profiles (49, 50).

Importantly, these cognitive differences occur in the context of preserved or even enhanced well-being, as indicated by lower depressive symptoms, better regulation of social rhythms, and higher quality of life. This suggests that the observed cognitive profile does not necessarily translate into functional impairment, but may instead reflect distinct cognitive strategies and underlying heterogeneity. Overall, the relationship between perceived energy levels and cognition in older adults appears complex and multidimensional, involving both differences in cognitive style and variability related to underlying subclinical conditions. However, given the cross-sectional design and the relatively small sample size, and the reliance on a single item- measure for group stratification, the present study should be considered strictly hypothesis-generating rather than hypothesis-testing (27, 51). Accordingly, these findings have primarily heuristic value.

Future studies should aim to disentangle these components through longitudinal designs and more refined phenotypic characterization, with particular attention to the identification of DYMERS-like subgroups.

## Limitations

This study should be interpreted in light of several limitations.

First, the relatively small sample size, particularly in the high perceived energy group, may limit the analyses’ statistical power and the generalizability of the findings. The reduced number of participants increases the risk of type II errors. Therefore, the results should be considered preliminary and warrant replication in larger samples. For the same reason, it was not possible to perform multivariate analyses to control the results for the main sociodemographic variables, due to the limited sample size. Indeed, a multivariate analytical model would not have been considered reliable under these conditions. Similarly, given the exploratory, heuristic, and preliminary nature of the findings, the calculation of effect size measures should be regarded as potentially misleading.

Second, the classification of high perceived energy was based on a single-item measure derived from the SF-12 questionnaire. Although this approach is supported by previous research suggesting its utility as a screening tool (16, 52), it may not fully capture the multidimensional nature of hyperactivity, which includes behavioral, cognitive, and temperamental components. Crucially, this stratification approach introduces a methodological circularity (part-whole overlap), given that Item 10 is an intrinsic component of the total SF-12 score utilized as a primary outcome measure for health-related quality of life. Consequently, the finding that individuals with higher perceived energy exhibit significantly better quality of life must be interpreted with strict caution, as the observed association is inherently confounded and potentially inflated by this mathematical overlap. Future studies should employ more comprehensive and validated instruments specifically designed to assess energy levels independently from broader quality of life outcomes.

Third, the cross-sectional design of the study does not allow for causal inference regarding the relationship between perceived energy levels and cognitive performance. It is therefore not possible to determine whether high perceived energy contributes to worsening cognitive performance over time or whether shared underlying mechanisms influence both. Longitudinal studies are needed to clarify the temporal dynamics of this association.

Additionally, although we attempted to exclude individuals with significant depressive symptoms and dysregulation of social rhythms, the possibility of residual confounding cannot be entirely ruled out. Other unmeasured factors, such as cardiovascular risk, lifestyle variables, or subclinical psychiatric features, may have influenced cognitive outcomes (53).

Finally, cognitive performance was assessed using a single screening instrument (ACE-R) (37). While this tool provides a broad evaluation across multiple cognitive domains, it may not capture the full range of performance nuances or domain-specific differences. Future research should incorporate more detailed neuropsychological batteries to obtain a more comprehensive characterization of the cognitive profiles associated with distinct energy profiles.

Despite these limitations, the present findings make a novel contribution to understanding the relationship between perceived energy and cognitive aging, highlighting the need for further investigation within a dimensional, integrative framework.

## Conclusions

The present study explores a complex and heterogeneous relationship between perceived energy levels and cognitive performance in older adults with preserved well-being and adequate social functioning.

Although no significant differences emerged in global cognitive performance, individuals with high perceived energy showed a consistent tendency toward lower scores across multiple cognitive domains, with significant differences in specific language and attention/orientation domains. This pattern suggests selective differences in cognitive performance rather than a generalized reduction.

Within this framework, one speculative possibility is that the substantial variability observed within the high-energy group may reflect the coexistence of different underlying profiles. Some individuals may represent a regulated and functionally advantageous high-energy condition, whereas others may present features conceptually consistent with subclinical forms of dysregulation. Importantly, the present study did not directly assess DYMERS, bipolar-spectrum characteristics, or related psychopathological constructs. Therefore, any reference to DYMERS-like profiles should be considered purely heuristic and hypothesis-generating. The observed variability may equally reflect differences in cognitive style, unmeasured individual characteristics, residual confounding factors, or sampling variability. Future studies employing multidimensional assessments of activation, mood regulation, biological rhythms, and temperament will be necessary to determine whether distinct phenotypic subgroups can be reliably identified within populations characterized by elevated subjective energy levels (13).

Importantly, these cognitive differences occur in the context of preserved or even enhanced well-being, characterized by lower depressive symptoms, better social rhythm regulation, and higher quality of life. This supports the view that high subjective energy may simultaneously represent a resource and a marker of heterogeneity, rather than a purely adaptive or maladaptive trait.

Overall, while there is an inherent gap between our sophisticated psychopathological framework and a narrow empirical measure, these findings raise a valuable question regarding energy as a primary mood dimension. Rather than showing a uniform pattern of decline, the substantial variability in cognitive performance observed within the high subjective energy cohort may index a crucial phenotypic heterogeneity. Specifically, some individuals within this healthy population may present a regulated, benign high-activation profile, whereas others might exhibit a more vulnerable or dysregulated profile. This interpretation should be regarded as speculative and hypothesis-generating. The observed variability may reflect the coexistence of different underlying profiles, although the present study was not designed to identify or characterize specific psychopathological subgroups. These trajectories could reflect, at least in part, different underlying cognitive styles.

Some cognitive styles in the literature have been associated with activation of the Behavioral Approach System (BAS). Specifically, findings indicate that individuals with bipolar vulnerability tend to exhibit greater reward sensitivity and heightened reactivity to signals of success, together with a strong drive toward goal attainment and elevated expectations of success (54, 55). While our single-item operationalization of perceived vitality obviously cannot capture formal hyperthymic temperament or mood activation, these psychopathological constructs serve as a broader conceptual backdrop to frame the dialogue around elevated levels of activation and energy. In this context, contemporary literature conceptualizes activation as a distinct clinical dimension spanning both subjective energy and objective behavioral drive (56). Broadening our perspective to these models helps contextualize our exploratory data within a dimensional mood spectrum, without implying any direct empirical correspondence between our single-item measure and formal bipolar phenomenology.

Taken together, these findings may point to distinct BAS-related cognitive-motivational configurations that could underlie variability in goal-directed behavior and decision-making. However, such interpretations remain tentative and should be regarded as hypothetical, requiring further empirical investigation.

Further research is needed to clarify the clinical significance of these patterns, particularly through longitudinal designs and more refined phenotypic characterization, to identify distinct subgroups and their respective cognitive outcomes.

## Data Availability

The raw data supporting the conclusions of this article will be made available by the authors, without undue reservation.
